# On the Connexion Between Morbid Physical and Religious Phenomena

**Published:** 1855-10-01

**Authors:** J. F. Denham


### ON THE CONNEXION BETWEEN MORBID PHYSICAL AND RELIGIOUS PHENOMENA.

No. 4 of a Series.

BY THE REV. J. F. DENHAM, M.A., F.R.S., &c.

In now proceeding to give classifications of the co-existing morbid physical and religious phenomena which have come under the writer's notice, it seems natural to commence with those relating to the department of faith, or religious belief; and consisting of scepticism or the incapacity of confidence in the first principles of religion, as one extreme; and of credulity, or the extravagance of religious belief, as the other. Beginning, then, with scepticism, I assume, as a maxim, that an acquiescent perception of all the primary truths, of at least natural religion, is the normal condition of the human mind:—or to use St. Paul's language, "that which can be known concerning God, is manifest *in* men, for God hath showed it to them, they know God, they perceive the righteous judgment of God;" so that, as he maintains, even the heathen were inexcusable in their abandonment of the true God, and idolatry; "because the invisible things of God are clearly seen, being intimated by the works of creation;"* or to use the words of Tertullian, "the human soul possesses the innate power of appropriating to itself, without any supernatural aid, all that may be known of the Divine being, by the works of nature;"† and, "for the first principles of religion, we need not appeal to a soul that has been bred up in a library and fed with academical notions, but to the soul that is simplex et rudis, et impolita, et idiotica, illa ipsa de compito, de trivio, de textrino," &c.‡ Not a few, also, of the most learned and orthodox Christians have in all ages maintained that a natural affinity exists between the native sympathies of the human soul and all the primary principles of even revealed religion. Thus, for instance, Tertullian says, the soul is naturally Christian. "O anima naturaliter Christiana!"§ When, then, the natural tendency of the mind to religious belief is found to be suspended, it seems reasonable, upon the principles laid down in the preceding papers, to attribute the phenomenon to bodily disorder affecting the mind, either as occurring in the course of nature, or as occasioned by vice. The following observations will, however, be directed to the subject of what may be called innocent scepticism, or that kind of scepticism which appears to result from innocently occasioned bodily disease.

The first case I give from my memoranda is of the lapse of faith under extreme physical exhaustion. A man, fair complexion, of an excitable temperament, ordinary powers of mind, common education; humane, friendly and cheerful in disposition; of good moral character in all respects, previous to his last illness, although in early life addicted to intoxication, and having, then, also, been under medical care for insanity, the exciting cause of which was, however, believed to have been his excessive attachment to a female whom he married after the recovery of his reason, and by whom he had several children; became at the age of forty-two afflicted with jaundice, succeeded by dropsy. Before his illness, and for many years, he was remarkable for the earnestness of his religious faith, for ecstatic singing, chiefly of Wesleyan hymns, and for excited prayer, for taking journeys to hear popular preachers and to attend "missionary meetings," and particularly for his vehement dislike to the Calvinistic doctrine of unconditional election to eternal life, and its consequent doctrine of reprobation.|| During the first part of his illness he

* Rom. i. 19—32. † De Testim. An. cap. 5. ‡ De Testim. An. cap. 1. § Apol. c. 17. || "Calvin's Institutes," chap. 23.

retained his religious belief, &c. He was tapped several times by a regular surgeon, and finally by an ignorant empiric, to whose care he waywardly resorted, and who cut an artery, thereby occasioning a great effusion of blood with the serum. *From the time of that operation* he exhibited a total repugnance of mind to all religious subjects whatever, could not endure prayer, would not allow the Scriptures to be read to him, argued, with what seemed in his case an extraordinary acuteness, against the benevolence of the Deity; maintained that he was reprobated of God, and predestinated from all eternity to everlasting destruction, or, that, if God had so reprobated and predestinated only one individual,—that individual was himself. He manifested also the most inveterate dislike to his wife, and even assailed her with the grossest abuse, could not endure the sight of his children, and suspected every one that approached him of some evil intention. His memory and judgment in his affairs remained comparatively unimpaired. He continued in this state for nearly two months; at last, and only a few minutes before he expired, he said, "Who can tell but that I may be saved?" The *post-mortem* examination disclosed a most extensive disorder of the liver, and the bowels enveloped with clotted blood. The surgeon remarked on the occasion, "This man was bled to death." His children all died early, of either diseased liver or fever.

Case 2, of a similar nature, but having a more pleasing termination, was of a lady, of fair complexion, excitable, and delicate constitution, devotedly attached to her husband and children, well-educated, intelligent, exemplary in conduct, and piously disposed, who, after a long and dangerous confinement, complained with distress of mind bordering upon delirium, of having no love or even reverence towards God, nor belief in the Scriptures, nor natural affection to her children, nor regard for her husband. She also maintained that her "spinal marrow had become severed, and that she had distinctly felt it part in sunder." After a residence of a few months at the seaside, receiving careful medical treatment, she completely regained all her religious confidence and correct feelings. Her delusion also subsided.

Case 3 is of a widow, about sixty years of age, dark complexion, sensitive feelings, imaginative mind, well-educated, humane, of excellent moral conduct, had had no children, was habitually dyspeptic. In early life she had imbibed high Calvinistic notions, had been accustomed to attend popular preachers of such notions, relied greatly on which she called her "experience,"—became, about fifteen months before her death, the subject of intolerable remorse of mind, along with visibly declining health, and her removal from the metropolis to a lonely country town—accused herself of having committed every sin, of being the author of all the natural and moral evil in the universe, and thought she was doomed to live till she had repaired it all; that she was a demon, that she killed every one that died, that she felt that she was killing them; fancied that she heard thieves in the house at all hours, or that the house was on fire; heard voices; pronounced "all her experience a delusion;" refused prayer, "because God had never been her God," or interrupted it with despondent expressions; exhibited other decisive symptoms of insanity: died without any mitigation of her state of mind. According to the opinion of her medical adviser, the digestive organs were totally impaired. It was said that insanity in lighter shades, of a similar nature, had shown itself in her mother.

Case 4 is of a younger female, mother of several children, delicate constitution, limited education, little native vigour of mind, devout, and of irreproachable conduct; during her long and fatal illness expressed a most comfortless state of mind in consequence of her total loss of faith, and of all regard to religion, prayer, &c. The utmost admission she could make, after every assistance had been rendered to alleviate her distress, was thus stated by herself: "I think that I have a wish to have a wish to believe, &c."

Case 5 is of permanent scepticism unattended with any *marked* indication of disease. A man forty-four years old, dark complexion, hypochondriacal temperament, uneducated but shrewd; brought up among the Wesleyans; violent when provoked, otherwise a man of good conduct:—working at one of those sedentary and manual trades whose followers furnish a large number of sceptics and fanatics—his mind always musing on abstruse topics, seemingly incapable of forming or retaining any conclusions, inclined to listen to instruction, and to speak on religious subjects without the appearance of guile or vanity. His naturally morbid temperament, and the want of sufficient mental occupation along with his handicraft, seemed to have combined in producing what Paley calls "a debility of mind that can trust to its own reasonings in nothing."

Case 6. A young man, florid complexion, sensitive, meek disposition, commercial education and pursuits, much respected for his moral conduct, had once been the subject of emotional piety, complained of incapacity of faith, of religious and moral indifference, hopelessness, inability to pray, &c. He was subject to habitual depression of spirits, seemed absent at times, and presented the appearance of determination of blood to the head. Continued so many years.

Case 7. An elderly man, who had been successful in business, the head of a large family, always respectable in moral conduct, of weak reasoning powers, emotional feelings, Calvinistic notions, extreme self-abasement, a zealous attendant on Calvinistic preachers, and at the meetings of religious societies of the same class,—after an epileptic fit retained the use of his faculties and his knowledge of general subjects, but manifested extreme suspicion of everybody, intense anxiety about money, along with a total indifference to all religious topics and objects, and even a forgetfulness of the religious phraseology which had once amply tinged his general conversation; became imbecile and died.

Case 8 is of a converted Jew, fair complexion, about thirty-two years of age, belonging to a sedentary and solitary occupation, intelligent, but not highly educated, amiable in disposition, and correct in morals; supported during his last and lingering illness (atrophy) by respectable Christians, who fully believed in his sincerity and good deportment. He retained every quality that entitled him to the support and esteem of his friends through many months of his disorder. During the last stage of it, which was accompanied with febrile symptoms, he disclaimed his adopted faith, and his faith in all religion. "Could not believe that he had deserved so much affliction, or that it could be needful, or useful to him. Could not believe in the goodness or providence of God." Declared that he had not been sincere in regard of his conversion; and that, though he had no real faith in any religion, he would die in the profession of that of his forefathers; denied that he had any need of repentance, exhibited great irritability and ill-temper, raved, blasphemed: delirium and death ensued. I have often seen a tendency in persons during their last illness, to return to the religious opinions in which they were bred, when different from those which they had adopted at a subsequent period: and what may, perhaps, be partly owing to the same causes, a resumption of their provincial *patois* and pronunciation, although it had been disused for many years.

Case 9 would include instances of persons of melancholy temperament, of sedentary occupations and habits, whose minds have habitually fallen, while working at their employments, into sombrous reveries of a religious nature. They have described their minds as "always working on such subjects;" and as, according to their belief, continuing to work on them during their sleep, because conscious of them as soon as they awoke in the morning. They have seldom seemed able or inclined to remove such a morbid state of mind by actual inquiry or instruction, their amount of information has appeared small, nor were they competent to state distinctly the causes of their mental uneasiness.

Case 10 would comprehend persons who have either entered upon theological inquiries in a state of ill health, or have pursued such inquiries so intensely as to induce disease. The progress of their malady has been marked by an increasing distrust of all tradition, a disregard to all authority, want of confidence in all common and intuitive principles, and an anxiety to examine the very foundations of all human belief and knowledge. The usual termination has been a return to health, and a discontinuance of their unregulated passion for inquiry, or an increase of disease, and of that passion, the decay of the faculties, and death.

The foregoing cases are given as *types* or representations of the chief *kinds* of religious scepticism attended with morbid physical phenomena, each of them, however, comprehending under it numerous diversities and modifications. I beg to offer the surmise that in most, if not all of such cases, religious belief, or at least the capacity of it, may still exist, but that the morbid physical feelings may disguise or distort it to the mind's own apprehension, or rather that those feelings may be mistaken by the mind for its own perceptions—that they are, in fact, simply cases of what may be called scepticism of the feelings. How far physical disturbances may suggest ideas, and even absurdly consistent trains of such ideas, may be seen in the extreme case of them, in which the patient *hears voices* uttering entire sentences, consisting of persuasions to evil deeds, or assailing him with abuse, and accusing him of crime, &c., depreciating the character and conduct of friends and relatives, blasphemous or obscene, but which from their being attended with fever or bodily disorder, and ceasing with its cessation, clearly indicate their physical origin. The endless yet regular repetition of the same things by such voices would seem to indicate their connexion with a disordered circulation. I have frequently met with such distressing cases, not, as far as I could learn, arising, as in the case of *delirium tremens*, from the abuse of spirituous liquors, but generally accompanied with deafness and the peculiar expression of the eye which indicates an oppression or a too high temperature of the brain. It is certain that morbid bodily states suggest corresponding ideas in dreams, and that "a natural indisposition and an imperfect sense of the beginning of a disease may vex the fancy into a symbolical representation; for so the man that dreamed he swam against the stream of blood had a pleurisy beginning in his side; and he that dreamed he dipped his foot in water and that it was turned to a marble, was enticed into the fancy by the beginning of a dropsy."* Is it then unreasonable to attribute to the action of bodily disease the same "symbolical representation" to the mind of morbid religious perceptions during the waking hours of the invalid, when his faculties are generally more or less in a dreamy condition?

In now proceeding to consider the opposite extreme—credulity, or extravagant religious belief—it may be premised that it is rarely found unassociated with a disuse, or distrust of the reasoning powers, both which are indications of bodily disease, (or infirmity,) or with a deficiency or neglect of mental culture and mental employment, which also tend to produce it, or with positive indications of diseased heart or brain. Its collateral symptoms are credulity respecting some or all other subjects, timidity, self-neglect, insensibility to moral obligations, and an instability of attachment to friends and other objects. It is also well known that vice of all kinds tends to produce general and even religious credulity. Vicious persons are often credulous and superstitious. Idolatry is throughout the Scriptures represented as associated with both vice and mental fatuity.

The following remarks will, however, be directed to the phenomena of credulity as associated with innocent bodily disease. The chief characteristics in such cases are,

1. Unsteadiness of religious opinions, &c., and a facility of passing rapidly to opposite extremes, from reverence to irreverence, and, what is more to be

* Bishop Jer. Taylor. Sermon 3, on Godly Fear. Part iii.

deplored, from a state of religious fervour to the commission of the grossest sins. The subjects of such mutations often complain of having an endless succession of the most diversified ideas and emotions. They are often charged with hypocrisy and folly by their more sober and discerning neighbours; but the interminable mutability and simulation of their physical feelings is the real cause of their apparent inconsistency and extravagance.

2. Credulity often assumes the shape of an undue tenacity for correct theological opinions, "right views," as they are termed; but attended with indications of its morbid physical origin, such as an inordinate anxiety respecting other subjects, and its subsidence with returning health. This tenacity often amounts to an absolute monomania in regard of certain doctrines, especially regeneration as dissociated from Baptism, Divine influence, Satanic temptations, sin, the difficulties of salvation, &c. Upon examination it will generally be found to consist of a mere unintelligent dismay, passion, or excitement, connected with some exaggeration of truth, or absurdity.

3. Credulity often manifests itself in a blind and uncontrollable regard for certain performances, such as the mere reading of the scriptures at particular times, or of a certain quantity of their contents; a mechanical observance of the Sabbath, ceremonies, &c. Prayer is particularly a subject upon which the morbid mind concentrates its endless and inactive anxieties: and instead of regarding and employing it simply as "asking those good things we have need of," such a mind can only conceive prayer to be genuine when inspired, or offered with a certain peculiar kind of feeling, or attended with a certain intensity of feeling, and may continue even for many prayerless years to endure its own inconsolable regrets for being denied the gift of such feelings, or for its own inability to command them.

The physical theory of credulity I would offer is, that owing to the inverted action of the mind, the physical feelings are the objects of its attention, and are either mistaken by it for its own perceptions, or originate its perceptions; and that according as these feelings are elated or depressed, or permanent or fluctuating, or strong or weak, so will be the phenomena of superstition—that credulity is, in fact, the credulity of the feelings, and that the particular modification it will assume depends greatly on the nature of the bodily disease.

The practical inferences from the foregoing sketch would seem to be, that no safe conclusion in regard of the existence of religious faith or of scepticism can be derived from the feelings; that persons of a morbid temperament should avoid the study of theology; that religious education and instruction should chiefly be addressed to the understanding, and that the exercise of a sound and enlightened judgment should never be relaxed in regard of all religious subjects and duties, &c.

Where morbid physical and religious phenomena of the foregoing kinds are developed, the removal of them depends on the cure of the bodily disorder from which they arise. But since in all such cases the existence of organic disease is to be more than suspected, a total prohibition of everything that excites or promotes its action should be enforced: such as introspection or the examination of the supposed religious phenomena of the mind; all conversation on the subject of religion, except with "a learned and discreet minister of God's word;" reading or hearing impassioned religious compositions, &c.; the attention should be restricted to a few first principles of belief and duty, and constant occupation, both mental and manual, or rational and improving amusement should be provided. The writer begs to add, as a general inference from the subject, the humiliating but salutary and charitable tendency of pathological studies, and the indispensable importance of them to the clergy; for such studies serve to show that religious principle may exist under at least a neutrality of appearances:—to acquaint us with the merely physical and even morbid origin of feelings which are too often admired and cultivated—to guide the faithful pastor in his efforts to promote the real welfare of his fellow-men, and to reconcile him to those discouragements which too often attend those efforts in proportion as they are wise and sincere.

---

### INSANITY AND DEMONIACAL POSSESSION.

